# The Origin and Evolution of the 18th Century Hospital Movement
*Previous articles appeared on December 13, 1913, January 17 and 31, February 14 and 28, 1914.


**Published:** 1914-03-14

**Authors:** 


### VI. THE ORIGIN AND EVOLUTION OF THE 18th CENTURY HOSPITAL MOVEMENT.*

In 1736 some gentlemen who had long observed the fruitless expense which attended the care of the sick poor in each distinct parish, the bad management and consequent waste of money in the administration of private charities, and the diminution in the contributions of the public who were becoming aware of these abuses, determined to take the necessary steps towards the erection at Winchester of a public hospital for the sick and lame. Although several workhouses had been established in various parts of the kingdom where medicines were dispensed, they recognised that the latter were generally given without the advice of physicians, and were only supplied to the poor of that particular district in which the workhouse was situated. Under the patronage of Dr. Alured Clarke, then Dean of Winchester, who was the principal promoter and supporter of the undertaking, a proposal for erecting a public hospital at Winchester upon the voluntary subscription principle was issued to the public.

To this a favourable reception was accorded, although certain objections were raised which necessitated a reconsideration of the whole design; which objections, and the answers returned to them, reflecting as they do the opinions of the time, throw a new and interesting side-light upon the evolution of the voluntary hospital movement.

"1. It was said that a workhouse is more wanted than a hospital. It may be enough to observe that whenever a better provision is made for the sick poor by Parliament this house will either cease or become part of that provision. The design now in hand of erecting public workhouses in every county is a strong argument in favour of the present scheme.

2. It was objected that the poor dislike anything with the appearance of constraint or removal from their families. But any who desire it shall be considered and received as out-patients. Experience tells that wherever a hospital has been erected the poor have esteemed it a blessing and have flocked to it with eagerness; for there they get free air, wholesome and proper diet, clean and constant attendance, the best advice, and *no more medicines than are necessary* [*sic*].

3. Others supposed that a hospital would occasion the resort of poor people to the city. But no person will be entitled to receive any medicine unless recommended by a contributor, and anyone, not inhabiting the city or suburbs, who asks alms may be incapacitated from hospital relief and treated as a vagrant.

4. Others said that there would not be objects enough in the country to answer the purposes of a public hospital. But they do not consider the great numbers that apply for relief in the winter, suffering from dropsical, rheumatic, paralytic, and scorbutic diseases, and slow and intermitting fever; and they do not consider the cases requiring surgical operation, which have to be removed by waggon to London (a three or four days' journey), after they have been fortunate enough to procure letters of recommendation and a promise of admission.

If such an institution has been found of such general use that it has been established in most of the provinces of France with the greatest success, as also in our own country, no further argument should be required.

1. Moreover, it is the only certain way of relieving the sick poor, because physicians and surgeons cannot conveniently attend at more places than one.

2. It is the most safe and eligible manner of doing so, because of the care and neatness, simplicity and regularity of diet given in a hospital.

3. The expense bears no proportion to that of assisting the poor at their separate homes.

4. It opens a channel for private charities long wanted; each subscriber can recommend a patient and knows that his subscription cannot be misapplied; whereas almost all private charities have been wasted by indolence, want of knowledge, and other mismanagement.

5. It is incapable of being abused or misapplied so as to make anyone sorry for subscribing, for even if some part were misapplied, the remainder would do more good in this manner than a much larger sum in any other. A thousand would be relieved here at less expense than a hundred in the ordinary way of alms-giving.

6. It is subject to no imposture that will not be discovered by the physicians and surgeons.

7. It prevents most of the evils common to the poor by giving a present support in sickness; it supplies all their wants more effectually than money, for even money cannot supply the instant relief here afforded in accidents, to which the poor are more subject than their betters.

8. It provides for the relief of multitudes unable to afford advice or physic, but now known as 'the poor,' because they do not come under the care of a parish or workhouse; they are the principal objects of this charity, since they are in present want though diligent and industrious, and thus form part of the useful and valuable classes.

9. It eases families of the burden of attending and providing for sick relations; these are relieved and comforted, and their families are at the same time able to earn their own support.

10. It preserves them from the ill-usage of ignorant quacks and impostors, who take advantage of their necessities, take their little money by selling them cheap (?) medicines, and destroy their health by want of honesty or skill.

11. It is of infinite use to all other persons, rich as well as poor, by furnishing the physicians and surgeons with more experience in one year than they could have in ten without it.

12. It is a most certain means of increasing the population, and saving a multitude of those who are lost by want of timely assistance; for one-third part of a labourers' earnings is so much clear gain to the public.

13. It enables parishes, when relieved of the burden of supporting the sick, to provide better for orphans and aged persons, whose numbers must gradually decrease by care of the sick, who will be enabled to educate their children and provide for their own old age.

14. It reduces the number of vagrants by depriving them of one of their most plausible reasons for begging from door to door under the specious pretence of sick relations or friends. Those who are idle and able to work will be obliged to do so when they are not supplied in the usual manner from the public and private charities, too often distributed without due regard.

* Previous articles appeared on December 13, 1913, January 17 and 31, February 14 and 28, 1914.

15. It is calculated to promote a spirit of religion and virtue; and, as a hospital is supplied with patients from all parts, that spirit will be gradually spread throughout a whole county. The regularity of manners, the removal from bad example, and the thoughts suggested in a house of mourning will improve them.

16. It is the most comprehensive of all charities. The sick are visited and relieved, the stranger taken in, the ignorant instructed, and the bad reclaimed. Present wants are supplied and future ones prevented. By easing families of the support of their sick, it feeds the hungry, clothes the naked, cherishes the industrious, and preserves a number of useful persons to the public good."

